# Davis’ Medical History

**Published:** 1850-05

**Authors:** N. S. Davis

**Affiliations:** Prof. of Physiology and Pathology in Rush Medical College


THE NORTH-WESTERN

MEDICAL AND SURGICAL JOURNAL.

Vol. III.] MAY, 1850. [No. 1.

### Part 1.—Original Communications.

### CHAPTER I.

*History of the Medical Profession from the first settlement of the British Colonies in America, to the year* 1783.—By N. S. Davis, M.D., Prof. of Physiology and Pathology in Rush Medical College.

[Copy Right Secured.]

The origin of the institutions of this, unlike those of most other countries, is involved in no obscurity; and is to be traced to no remote chivalric or feudal age. On the contrary, they have all had their birth since the middle of the seventeenth century; and been exposed to the full gaze of an enlightened world.

This, together with the fact, that they have not only had their beginning, but their maturity, thus far, among the most enterprizing people, and under the influence of the most liberal government on earth, renders every thing connected with their history, doubly interesting and important, as affording the fairest illustrations of human progress, and consequently the most valuable lessons of human experience.

The Medical Profession in the United States, and, indeed, throughout the civilized world, constitutes an important part of society; for while on the one hand, its ranks can boast, not only, of names of the highest eminence in every department of science and literature, but can also claim to be equal with the foremost in every enterprise for extending human knowledge, and ameliorating human suffering; its free access to the homes and firesides of all classes, gives it a moral and social influence of the most potent character. And in no part of the world is this influence more extensively or happily felt, than in this country, where the absence of all hereditary distinctions and privileged orders, leaves learning and virtue free to assume their own native eminence.

As far as can now be ascertained, but very few regularly educated physicians embarked with the first colonists that planted themselves in the wilderness of America. We are told by Dr. S. W. Williams, of Deerfield, that Dr. Samuel Fuller, a regularly educated physician and highly esteemed man, accompanied the first emigrants who landed at Plymouth in 1620. He was a faithful and devoted practitioner, and died of an infectious fever at Plymouth in 1632. The name of Dr. Russell is also mentioned as a companion of Captain Smith, in his survey of Chesapeake Bay in an open boat, in 1608. But the fact that Smith was obliged to return to Europe the very next year, to procure surgical aid, on account of an injury to his hand, "there being none to be had in the colony," shows that Dr. Russell's stay was short, or else he was not a man skilled in his profession. These are the only names that we can find mentioned among the first settlers, either at Jamestown, Plymouth, or New-York. The fact that there were either very few physicians among the early colonists, or that they were poorly prepared to discharge their responsible duties, is further corroborated by the almost total neglect of those sanitary regulations so necessary to preserve their health, and the consequent great mortality that took place in all the colonies during the first few years. A surgeon is mentioned as on board one of the ships sent to aid

Capt. Mason in his expedition against the Pequoit Indians in 1637, but whether he belonged to the colony, or the ship merely, is not known. In 1649 we find a law passed by the Massachusetts Colony, forbiding "Chirurgeons, Midwives, Physicians, or others, to exercise, or put forth any act contrary to the known rules of art, in each mystery and occupation, to exercise any force or violence or cruelty, upon or toward the body of any, whether young or old (not even in the most desperate cases) without the advice and consent of such as are skillful in the same art, (if any such may be had,) or at least, of the wisest and gravest there present, and consent of the patient or patients, if they be *mentis compotes*, much less contrary to such advice and consent, &c." This provision is sufficiently indicative of the condition of the profession, at this early period, in the New-England States.

Indeed, with the exception of the two Governors Winthrop, one of Massachusetts and the other of Connecticut, and a few of the clergy, we find no names of even respectable attainments in the profession, during the first half century after the commencement of our colonial existence.—The younger Winthrop, not only practised medicine extensively, but was also a member of the Royal Society of London, to which he made several respectable communications. And even so late as 1753, we are told by the Independent Reflector, a paper then published in the city of New-York, "that it (the city of N. Y. then containing about 10,000 inhabitants) could boast of more than *forty* gentlemen of the faculty, the greatest part of whom were mere pretenders to a profession of which they were entirely ignorant; and convincing proofs, of their incapacity were exemplified in their iniquitous practices. The advertisements they pnblished proved them ignorant of the very names of their drugs" &c., &c. Dr. Nicholas Romaine, in his annual address before the New-York State Medical Society, (in 1811) speaking of this early period, says: "It would be *painful* to intrude on your notice, the

*humble* condition of medicine which seems to have existed for more than a century after the first settlement of this State.—It could only consist of a statement of the *arts* and *intrigues*, by which the practitioners of physic succeeded in advancing their *private interests* and professional emoluments." The reasons for such a state of things, at this period, are obvious,---there being no medical schools in the colonies, or any institutions for the instruction of medical students, the only sources of supply consisted in emigrations from the mother country, or in the sending of native young men to the hospitals and colleges of Europe.

The Circumstances of the country during the whole of the first century were such, that no man already established in practice on the other side of the Atlantic, would think of leaving it for the hardships, the poverty, and wilds of America; while the absence of all those Medical Societies and Institutions, which constitute so powerful an object to the aspiring ambition of the thoroughly educated student, equally prevented this class from resorting hither. Hence, as a general rule, only those physicians who had failed to obtain a practice at home, or were too conscious of their own unfitness to make the attempt, emigrated to America. On the other hand, the great expense attending the education of young men belonging to the colonies, in the Medical Institutions of Europe, operated as an equal barrier to this source. Thus it was, that while persecution filled the clerical ranks of the colonies with men of the deepest piety, and the most varied learning, and the patronage of the crown induced a full supply of legal talent, the profession of medicine sunk to a comparatively low state.

In New-England, the greater number of those who practiced medicine were priests, whose medical knowledge was chiefly derived from the writings of Hippocrates, Galen, Aretæus, &c., which they had read during their collegiate education in Europe. Some of the non-conformist clergy, however, who were persecuted or silenced in the Old World, went through with a regular course of medical studies before leaving home, and afterwards became exceedingly useful, both as physicians and preachers, among the colonists here. Of this number, were Drs. Giles Firmer and John Fisk; the first arrived in New-England 1633, and the last in 1637. Both lived to an advanced age, and were highly respected. Dr. Thomas Thatcher, also came about the same time; and his pamphlet entitled "A Guide in Small-Pox and Measles," published in 1677, is said to be the first medical publication in America. About the middle of the seventeenth century, Drs. Bellingham and Henry Saltonstall, after receiving a general education at Harvard College, went to England, where they completed a full course of medical studies, received the degree of M.D., and returned to practice their profession in the Massachusetts Colony. These were the first young men of whom we can get any account, who left the colonies to obtain a regular medical education in the mother country, for the purpose of practicing here.

But during the latter part of the first half of the eighteenth century, a new era began to dawn on the medical profession in America. It was during this period that we find several young men of the highest order of native talent, after receiving a thorough course of instruction in the medical institutions of Great Britain and the Continent, returning to practice their profession in the New World. Among these we find the names of Dr. Zabdiel Boylston and Dr. James Lloyd of Massachusetts; Lieut. Gov. Colden and Dr. James Ogden of New-York; Drs. John Morgan, William Shippen, Jr., and Benjamin Rush, of Pennsylvania; Drs. John Mitchell and Thomas of Virginia, and Dr. Lining, of South Carolina—names, that will ever remain as ornaments to the medical profession in America.

During this period, other circumstances also occurred which aided very much the advancement of the profession.—Wars between England and France, were carried on, with only short intervals of peace, during the greater part of the eighteenth century. And so early as 1690, hostilities commenced between New-York and New-England on the side of England, and the French settlements in New-Brunswick and Canada. From this time to the final subjugation of the French colonies in 1763, many expeditions were sent from England to aid the colonists, and each was accompanied by a well appointed Medical Staff. These being almost constantly in the colonies, with the military hospitals which necessarily accompanied the movements of regular armies, supplied the place, in some degree, of medical schools, and doubtless excited the ambition of many of the young men, who, during the latter part of this period, spent some time in Europe and returned to become the founders of Medical Science in America. What Dr. Nicholas Romaine says of the influence of these, on the medical profession of New-York, is equally true of New-England, Pennsylvania, and Virginia. In his annual address to the Medical Society of the State of New-York in 1811, to which we have already alluded, Dr. Romaine says: "The war which effected the conquest of Canada, was, perhaps, the first circumstance which materially improved the condition of medicine in this State, (New-York). The English army employed for that purpose, left Europe accompanied by a highly respectable Medical Staff, most of whom landed in the city of New-York, and continued some years in the neighboring territories, affording to many young Americans opportunities of attending the military hospitals, and receiving such professional instruction as gave them afterward consideration with the public. The physicians and surgeons of the Anglo-American army, gained the confidence of the public, by their superior deportment and professional information. The military establishment in this State, after the Canadian war, required medical and surgical attendants; so that the people had the benefit of their advice.

In this manner a new order of medical men was introduced into the community."

It was doubtless owing to the increased attention to medical science and practice, produced by these causes, that led the colony of New York to make the first effectual attempt to regulate the education and practice of the profession by legal enactment. We have already quoted from the Independent Reflector of 1753, a paper which not only exposed the ignorance and pretensions of the profession, but appealed with boldness and energy to the people for a remedy. And some of the arguments presented, would afford useful lessons for many legislators of the present day.

It was not until 1760 that the General Assembly of New-York ordained that, "no person whatsoever should practice as Physician or Surgeon, in the city of New-York, before he shall have been examined in physic and surgery, and approved of and admitted by one of His Majesty's Council, the Judges of the Supreme Court, the King's Attorney General, and the Mayor of the city of New-York for the time being, or by any three or more of them, taking to their assistance for such examination, such proper person or persons as they in their discretion shall see fit."

Such candidates as were approved received certificates, conferring the right to practice physic or surgery, or both, throughout the whole province; and a penalty of five pounds was prescribed for all violations of this law. Its restrictive operation, however, was confined exclusively to the city and county of New-York; and with the exception of a similar enactment in the colony of New-Jersey in the year 1772, it was the only attempt to regulate the qualifications and practice of physicians, by any of the colonial governments, previous to the Revolutionary War.

But the causes to which we have alluded, had already introduced into the profession many young men, possessed not only of sound learning, but also, of a laudable ambition for the honor and advancement of their favorite calling. Among these none were more zealous in the cause of medical education than Drs. John Morgan, and William Shippen, Jr., of Philadelphia, and Drs. Samuel Bard, Peter Middleton, and John Jones, of New-York. Although many private and temporary hospitals had been established in the several colonies, for the accommodation of seamen, and the reception of patients for inoculation, the first permanent institution of the kind was established in Philadelphia in 1752, and was aided by a grant of 2,000*l.* from the Colonial Assembly. Its establishment was owing to the suggestions and exertions of Dr. Thomas Bond, who became its superintendent, and we believe, the first regular *Clinical* Lecturer on Medicine in America. In 1762 Dr. Wm. Shippen returned from his studies in Europe, to settle in his native city, Philadelphia, and the same year commenced a course of Lectures on Anatomy, to a class of twelve students. He continued the same course during the two following years, and in 1765 was joined by John Morgan, M.D., who had just returned from an honorable completion of his medical studies in the schools of Edinburgh. Dr. Morgan soon delivered a public discourse on "the Institution of Medical Schools in America," before the Trustees of the University that had been established in Philadelphia, and proposed a plan for teaching the various branches of Medical Science. His views were warmly seconded by the Board of Trustees, who soon after appointed Dr. Morgan Professor of Theory and Practice of Medicine, and Dr. Shippen Professor of Anatomy and Surgery. The remaining chairs were not filled until 1768, when Dr. Adam Kuhn, was chosen Professor of Botany and Materia Medica; and in the following year Dr. Benjamin Rush returned from Edinburgh, and was appointed to the Chair of Chemistry. Thus organized, the first Medical School in America continued its annual courses of instruction to an increasing number of students until 1777, when it was suspended by the Revolutionary War. To give a still better idea of the objects and Regulations of this school, and the liberal views of its founders, we will quote the following rules in regard to studies and qualifications from a printed circular, addressed by Dr. John Morgan to the inhabitants of the West Indies in 1772, viz:

*For a Bachelor's Degree in Physic.*

"I. It is required that such students as have not taken a degree in Arts, shall, before admission to a degree in Physic, satisfy the Trustees and Professors of the College, concerning their knowledge of the *Latin* tongue, and in such branches of *Mathematics*, Natural and Experimental *Philosophy*, as shall be judged requisite to a *Medical Education.*

"II. Each student shall take at least one course in *Anatomy*, *Materia Medica*, *Chemistry*, the *Theory* and *Practice* of *Physic* and *Clinical Lectures*, and shall attend the practice of the *Pennsylvania Hospital for one year;* and then may be admitted to a public examination for a *Bachelor's* Degree in Physic, provided that on previous private examination by the *Medical Trustees* and *Professors* and such other Trustees and Professors as choose to attend, such student shall be judged fit to undergo a public examination.

"III. It is further required that each student, previous to obtaining a Bachelor's Degree, shall have served a sufficient apprenticeship to some respectable Practitioner in Physic, and be able to make it appear that he has a general knowledge in *Pharmacy.*"

*For a Doctor's Degree in Physic.*

"I. It is required that at least three years shall intervene from the time of taking the Bachelor's Degree, and that the *candidate* be full *twenty-four* years of age, and that he write and defend publicly in college, a *Latin Thesis* or dissertation on some disease, or other useful Medical topic, which shall also be printed at his own expense.

"II. The above scheme of a *Medical Education*, is upon as extensive and liberal a plan, as in any of the most respectable European Seminaries, and the utmost care is taken to render the Degrees *real marks of honor*, the marks of only distinguished learning and abilities. A good *medical education* is here looked upon as not the least useful part of Science. As it is both a noble and extensive branch of learning of itself, and of the utmost importance to the health and welfare of society, 'there cannot therefore be too much encouragement given to a full and regular attainment of it.' 'Tis, on this account, one *great object* in the Philadelphia College, and intended to put the practice of physic in America in general, upon a respectable and beneficial footing."—[See "The Address and Representation of Dr. John Morgan of the College of Philadelphia, in behalf of the Seminary, dated 1772,"]

Such were the views and objects of the founders of the first Hospital and College, in America. The rules quoted were adopted by the College on the 12th May, 1767, and continued in full force until the operations of the College were suspended by the occupation of Philadelphia by the British in 1777. During this period the Degree of Bachelor of Physic was conferred on about thirty young men, and a reasonable degree of prosperity seems to have attended the College. But while these things were being done in Philadelphia, the Profession in New-York were not idle. The zealous efforts of Drs. Bard, Middleton, and others, aided doubtles by a spirit of rivalry with Philadelphia, effected the organization of the Society of the New-York Hospital, and procured for it a charter from the Colonial Government in 1777. The first building was destroyed by fire in 1772 which, together with the occupation of New-York by the British army, prevented the completion of another, until 1791. when the first patients were received. Their efforts to establish a Medical College in connection with King's College, which had been established in the city several years previous, was, however, attended with better success. A full Medical Faculty was organized in 1768, composed of Samuel Clossy, M.D., Prof. of Anatomy, John Jones, M.D., Prof., of Surgery, Peter Middleton, M.D., Prof. of Physiology and Pathology, James Smith, M.D., Prof. of Chemistry and Materia Medica, John V. B. Tennent, M.D., Prof. Midwifery, and Samuel Bard, M.D., Prof. of Theory and Practice of Physic. The first courses of lectures were given in the winter of 1768–9, at the close of which, the degree of *Bachelor* in Medicine was conferred upon Samuel Kissam and Robert Tucker; and in the year following, the same gentlemen, received the higher degree of *Doctor* in Physic. These are stated by Dr. Beck, in his "History of American Medicine before the Revolution," to be the *first* Medical Degrees conferred by the schools in America.* But this is probably a mistake, as we find in a historical sketch of the College in Philadelphia, published in 1836, the names of ten students who received the degree of *Bachelor* of Physic, at the Commencement in that College, in June 1768; and a number of others were annually thereafter, honored with the same degree, until the operations of the School were suspended by the vicissitudes of war. During this period very few seem to have applied for the higher degree of *Doctor* in Medicine, either in Philadelphia or New-York. We have no means of ascertaining what rules were adopted for the government of the college in New-York; or what were the requisites for the graduation of medical students. It is certain, however, that the prosperity of the school was not proportioned to the known respectability and learning of the Professors.

Thus, we are told by Dr. N. Romaine, that in 1774, six years after its first organization, only "about 25 persons attended the Anatomical Lectures, some of whom were students from the West Indies." This want of prosperity has been attributed to the conduct of the governors of the College, but with what justice, we are entirely unable to judge. The

*See Transactions of the Medical Society of the State of New-York.—Vol. 5, pp. 141.

medical books then in general use, were the writings of Sydenham, Boerhaave, Van Swieten, Mead, and Huxham; the Physiology of Haller; the Anatomy of Cowper, Cheselden, and Munro; the Surgery of Sharp, Le Dran, and Pott; the Midwifery of Hunter and Smellie; and the Materia Medica of Lewis. But though the works then in general use were all of European production, yet medical literature had by no means been neglected by the profession in America; as the following list will show, viz:

"A Brief Guide in Small-Pox and Measles;" by Thomas Thatcher, of Massachusetts, published 1677. "Some Account of the New Method of Receiving the Small-Pox by Engrafting or Inoculation;" defending the practice, by Benj. Colman, of Boston, published in 1721. "An Account of the Climate and Diseases of New-York;" by Cadwallader Colden, of New-York, published in 1720. "An Historical Account of the Small-Pox Inoculation in New-England;" by Dr. Zabdiel Boylston, of Boston, published in 1727. "On the Method of Practice in Small-Pox in 1730;" by Nathaniel Williams, of Massachusetts. "A Treatise on Pharmacy in 1732;" by William Howard, of Mass. An Essay on Fevers, by Dr. John Walton, of Boston, in 1732. "The Practical History of a New Epidemical Eruptive Miliary Fever, with an Angina Ulcusculosa, which prevailed in New-England in 1735–6;" by Dr. William Douglass, of Mass., published in 1736. "An Essay on the Polygala Seneka;" by Dr. John Tennent, of New-York, published 1736. "An Essay on Illiac Passion;" by Dr. Thomas Cadwallader, published 1740. "An Essay on the Causes of the Different Colors of People in Different Climates," and "Letters on the Yellow Fever of Virginia;" by Dr. John Mitchell, of Virginia, published 1743. "The Flora Virginia;" by Dr. John Clayton, of Virginia, published 1743. "A very valuable account of Statistical Experiments concerning Excretions of the Human Body;" by Dr. John Lining, of South-Carolina, published in 1743. About the same time Dr.

Cadwallader Colden, of New-York, published "Observations on the Fever which prevailed in the City of New-York in 1741 and '42;" also, "On the Virtues of Great Water Dock;" and a "Sore Throat Distemper which prevailed Epidemically in this country in 1735." "A description of American Yellow Fever," was published by Dr. John Lining in 1753; and a paper on "Opisthotonos and Tetanus;" by Dr. Lionel Chalmers, of South-Carolina, published in 1754. "An Essay on the Use of Bark in Scrofulous Cases," was published by Dr. Thomas Bond of Philadelphia in 1759; also, an Inaugural Dissertation, entitled "Tentamen Medicum de Puris Confectione, Edinburgh, 1763," by Dr. John Morgan. Dr. Shippen's Inaugural Thesis, entitled "De Placentæ Cum Utero Nexu," was published in 1760; and during the same year, Dr. John Bard, of New-York, published a case of "Extra Uterine Fœtus," and an "Essay on the Nature and Cause of the Malignant Pleurisy," which prevailed on Long Island in the winter of 1749. In 1771, Dr. Samuel Kissam published his Inaugural Dissertation on the "Anthelmintic Virtue of the Phaseolus Zuratensis Siliqua Hirsuta, or Cowhage." A valuable work on the "Treatment of Wounds and Fractures," was published by Dr. John Jones, in 1776. And the same year Dr. Lionel Chalmers published his able work on the "Weather and Diseases of South Carolina." "An Essay on Fevers," had been published by the same author in 1768.

Many of these productions are characterized by a variety of learning, an accuracy of observation, and an originality of thought, which do credit to any age or country. Thus, Dr. Lining's "Description of American Yellow Fever," stands to the present day, unrivalled for its accuracy and minuteness of description ; and Dr. Douglass, in his "History of the New Epidemical Fever" of 1735--6, not only gives the best account of that fatal disease, but also enjoys the honor of first suggesting the use of Calomel as a remedy. Dr. Clayton's "Flora Virginia" attracted so much attention that it was republished at Leyden, 1762; and very few papers exhibit a greater degree of Philosophical acumen and learning than Dr. Mitchell's on the "Causes of the Different Colors of Different People." The Work of Dr. Chalmers on the Climate and Diseases of South-Carolina, in two volumes, is particularly worthy of attention, as well as that of the same character concerning New-York, by Dr. Colden. And the credit of originality, in promulgating the true doctrine in regard to the formation of *Pus*, though usually ascribed to Dr. John Hunter, undoubtedly belongs to Dr. John Morgan, who, in his inaugural thesis, maintained with great ability the doctrine that *Pus is a Secretion.* This is fully and honorably acknowledged by Dr. James Curry, lecturer at Guy's Hospital, so early as 1817.

Nor is this the only instance of fair claim to originality, which has been appropriated elsewhere. The use of Mercury, in the treatment of Inflammatory diseases and Eruptive Fevers had its origin with Dr. Douglass, of Boston, in 1736.—The preparation used was Calomel. But to Dr. Jas. Ogden, of Long Island, are the profession indebted for pointing out more systematically the indications for its use and the manner of using it. He used it extensively, and with the happiest effects, in the Angina Maligna of 1749; and its use was rapidly extended to the treatment of nearly all the Phlegmasia.—Again, to Dr. Richard Bayley, are we indebted, for first pointing out the true inflammatory nature of the Cynanche Trachealis, or Croup, and the great utility of Blood-letting and Antimony in its treatment. Yet all of these have been often set forth, as originating on the other side of the Atlantic.

Dr. James Lloyd, of Mass., and Dr. Wm. Shippen, of Philad. were the first regular and successful practitioners of Midwifery in this country. The one settled in Boston in 1754, and the other in Philadelphia in 1756; and to their skill, boldness, and decision of character, are we indebted for the rescue of that most delicate and important branch of practice, from the hands of ignorant and credulous females.

Although a consideration of Medical Practice, does not come strictly within the scope of our present work; yet an occasional glance at this, and the character of diseases prevalent at different periods of time will be both interesting and profitable. Indeed, a carefully written history of diseases, their variations of type and severity, in connection with the prevalent modes of medical practice, and the ever varying customs of society, would be of great value to the profession. It would explain the origin of many theories and systems in medicine, and reconcile much that now seems discordant and contradictory in our medical literature.

Thus says Dr. Rush :—"The success of nature in curing the simple diseases of Saxony laid the foundation for the *Anima Medica* of Stahl. The endemics of Holland led Dr. Bœrhaave to seek for the causes of all diseases in the *fluids*. And the universal prevalence of diseases of the *nerves* in Great Britain, led Cullen to discover their peculiar laws and to found a system upon them—a system, which will probably last till some new diseases are let loose upon the human species, which shall unfold other laws of the animal economy."

During the greater part of the period included in this chapter, the Humoral doctrines of Boerhaave held an unlimited sway over the minds and practice of physicians, both in this country and Europe. Endemic and Epidemic Fevers prevailed frequently, in nearly all the colonies, and sometimes produced great destruction to life. This was the case, to some extent, even among the Indians, before white settlements were formed. Thus, we are told by Dermer, Mather, and Gookin, that in 1618–19, a pestilential fever prevailed among the Indians in New-England, with such severity that whole tribes were nearly destroyed, and in some places, in 1620, their dead bodies were found unburied, putrefying in the sun. The first colony of Pilgrims that landed at Plymouth in 1620, suffered dreadfully from sickness during the few following years. And in 1632–33–38, a pestilence resembling the Yellow Fever, prevailed extensively in the New-England settlements, which, with the Small-Pox, cut off many of the inhabitants. Dr. Rush tells us that between the years 1760 and 1766, Intermitting, Bilious, and Yellow Fevers were common in Philadelphia and its environs; that Influenza was epidemic in 1761, and the Malignant Sore Throat in 1763. He also states that deaths were "common between the 50th and 60th years of life, from gout, apoplexy, palsy, obstructed livers, and dropsies."

This last paragraph is worthy of a moments consideration. Why were these diseases so frequent and fatal, when thirty years after the same author tells us they were comparatively rare in the same city? A very satisfactory answer to this question will be be found in the habits of the citizens at the two periods referred to. Alluding to these habits during the period intervening between the years 1760 and 1766, Dr. Rush says, "the diet of the inhabitants of Philadelphia, during those years, consisted chiefly of animal food. It was eaten, in some families, three times, and in all, twice a day. A hot supper was a general meal. To two and often three meals of animal food in a day, many persons added what was then called 'a relish,' about an hour before dinner. It consisted of a slice of ham, a piece of salted fish, and now and then a beef-steak, accompanied with large draughts of punch or toddy. Tea was taken in the interval between dinner and supper. In many companies, a glass of wine and bitters was taken a few minutes before dinner, in order to increase the appetite. The drinks with dinner and supper, were punch and table beer. Besides feeding thus plentifully in their families, many of the most respectable citizens belonged to clubs, which met in the city in the winter, and in its vicinity under sheds, or the shades of trees, in the summer, once and twice a week, and in one instance every night.

